# A Novel Modeling Approach for Plastics Melting within a CFD-DEM Framework

**DOI:** 10.3390/polym13020227

**Published:** 2021-01-11

**Authors:** Alptekin Celik, Christian Bonten, Riccardo Togni, Christoph Kloss, Christoph Goniva

**Affiliations:** 1 Institut für Kunststofftechnik, University of Stuttgart, 70569 Stuttgart, Germany; christian.bonten@ikt.uni-stuttgart.de; 2 DCS Computing GmbH, 4020 Linz, Austria; riccardo.togni@dcs-computing.com (R.T.); christoph.kloss@dcs-computing.com (C.K.); christoph.goniva@dcs-computing.com (C.G.)

**Keywords:** simulation, modeling, CFD, DEM, melting, melting apparatus, extrusion

## Abstract

Existing three-dimensional modeling approaches to single-screw extrusion can be classified according to the process sections. The discrete element method (DEM) allows describing solids transport in the feed section. The melt flow in the melt section can be calculated by means of computational fluid dynamics (CFD). However, the current state of the art only allows a separate consideration of the respective sections. A joint examination of the process sections still remains challenging. In this study, a novel modeling approach is presented, allowing a joint consideration of solids and melt transport and, beyond that, the formation of melt. For this purpose, the phase transition from the solid to liquid states is modeled for the first time within the framework CFDEMCoupling^®^, combining CFD and DEM by a novel melting model implemented in this study. In addition, a melting apparatus for the validation of the novel melting model is set up and put into operation. CFD-DEM simulations are carried out in order to calculate the melting rate and are compared to experimental results. A good agreement between the simulation and experimental results is found. From the findings, it can be assumed that the CFD-DEM simulation of single-screw extruder with a joint consideration of the feed and melt section is feasible.

## 1. Introduction

Single-screw extrusion is one of the most widely used production processes in the plastics industry. Semi-finished products such as tubes, hoses, foils, plates, and geometrically even more complex profiles can be produced [1,2]. Hence, single-screw extrusion is still the subject of current industrial and fundamental research. In recent years, computer-aided modeling of single-screw extrusion and research for new simulation approaches has become increasingly important. In general, the existing studies can be subdivided into investigations of the feed and the melting section. First attempts to describe mathematically the transport of solids in the feed section in one dimension go back to Darnell and Moll [3] and Schneider [4], who made the basic assumption that the interparticular friction is greater than the friction between particles and walls. Schneider [4] formulated the first equations for calculating flow rate and pressure build-up. Over the years, the first approaches were further developed in [5,6,7], until Schneider [8,9] suggested the insertion of axial grooves in the feed section in order to convey independently of pressure. Further studies [10,11,12] concentrated on the modeling and calculation of grooved feed sections.

In addition to Potente and Pohl [13], Moysey and Thompson [14] were pioneers in 2004, when they applied for the first time a three-dimensional numerical approach, the so-called discrete element method (DEM), to a smooth feed section. This method has the advantage that each granule and its interaction with surrounding particles and walls can be mapped individually. However, potentially higher computing times are disadvantageous. Over time, the results obtained by Moysey and Thompson [14,15] and the DEM itself proved to be very promising for calculating the feed section. Since then, further works such as Schöppner et al. [16,17] and Celik and Bonten [18] focused on the application of DEM. In addition, Leßmann [19,20] investigated a smooth extruder with rotational speeds up to 900 min^−1^ and considered axial forces acting on granules.

The DEM also proved suitable for modeling the transport of solids in twin-screw extruders. Interesting applications can be found in Carrot et al. [21] and Potente et al. [22]. Besides the solids transport itself, Schöppner [23] also modeled the heating of granules along a twin-screw extruder.

However, despite the valuable work done in recent years, the models are partly dependent on empirical model parameters. What is much more important though is that the investigation by means of DEM is always limited to the feed section, since the phase transition during melting cannot be modeled. As a result, the melting section, limited by the available numerical simulation possibilities, is considered largely separate from the feed section.

Initial investigations of the melting behavior go back to Maddock [24], who stopped an extruder during operation, let it cool down and then pulled the screw out of a barrel. He found that a melt film forms between a granular bed and a cylinder wall. Based on these investigations, Tadmor [25,26] developed a mathematical model to describe the melting behavior. Donovan [27] introduced extensions in terms of heating a granular bed along the screw, whereas Pearson [28] later assumed a nonconstant melt film thickness. Potente [29] also introduced analytical equations based on the investigations of Tadmor [25,26] to calculate the melting behavior.

Besides the analytical–empirical approaches mentioned so far, Viriyayuthakorn and Kassahun [30] pursued the aim to describe the melting process by means of conservation equations and equations of state, in order to investigate a model that is not limited to materials and process parameters. The melting process is not described by a phase change, but rather by the associated change of material properties. They chose the specific heat capacity as a decisive parameter. However, an experimental validation was not carried out. Altinkaynak’s [31] approach, based on the finite element method (FEM), was also basically developed out of this idea. However, Altinkaynak [31] chose the viscosity that changes during melting as a parameter. For the solid state, the viscosity is assumed to be infinitely high and decreases with increasing temperature. Hopman et al. [32] chose an approach based on the finite volume method (FVM) with a similar idea.

A very good overview of the basics of global modeling of single-screw extrusion and the existing modeling approaches can be found in Wilczyński et al. [33]. The authors concluded that a coupled approach via Computational Fluid Dynamics (CFD) and DEM for the holistic modeling of single-screw extrusion proves promising.

However, it is also required that a suitable melting model can precisely describe the phase transition. Karrenberg et al. [34] also criticized about the lack of a modeling approach combining CFD and DEM.

The present work resolves this aspect. The current state of research leads to the conclusion that the holistic modeling of single-screw extrusion is only possible under separate consideration of the respective feed and melting section. In the authors’ view, this is because until a few years ago there was simply no simulation tool that allowed describing the interaction of particles and fluids in three dimensions. Modeling the coexistence of several phases remains a challenge to this day, which is reflected in the fact that solid and liquid states are considered decoupled from each other.

Furthermore, from the authors’ point of view, a suitable melting model that describes the phase transition of plastic granules is an important aspect but is missing, thus enabling the holistic, three-dimensional simulation of the feed and melting sections to be necessary.

Therefore, the primary goal of this work is to explore a novel melting model for plastics to describe phase transitions. This melting model is to be implemented into the simulation software CFDEMCoupling^®^ [35]. This is an open-source coupled CFD-DEM framework which extends OpenFOAM^®^ [36] to include a coupling with the DEM software LIGGGHTS^®^ [37], which stands for LAMMPS improved for general granular and granular heat transfer simulations.

In order to ensure reliable results, the aim is also to validate the melting model experimentally. For this purpose, a melting apparatus will be set up and put into operation. This apparatus reproduces similar physics appearing in a real single-screw extruder. With the help of the experimentally obtained data, conclusions can be drawn about the reliability of the melting model. The overriding and long-term goal is to apply the newly researched simulation method to single-screw extrusion in the sense of holistic simulations.

## 2. Novel Modeling Approach

The new melting model stands out from the current state of the art, as the melting behavior is not represented by a temperature-dependent change in the dynamic viscosity or the specific heat capacity (see [30,31]). The novelty of the melting model is given by a general CFD-DEM approach [38], which couples the so-called volume of fluid method (VOF) [39,40] with the DEM. The cutting-edge features of this model include the physical interaction between three phases (air, solid, and molten particles), the heterogeneous temperature distribution within a particle and the particle melting representation. Regarding the latter feature, a decrease of the radius of a DEM particle as a function of the temperature during the melting process is explicitly represented. The main requirement of the model is to describe the phase change of plastic granules under consideration of heat conduction, convection, particle heating due to friction, and the resulting mass change.

In the following, the focus lies in the description of the modeling approach. First, the existing basic equations of the DEM are introduced. Then, all extended model equations including the novel melting model are presented.

### 2.1. DEM Approach

For the description of particle–particle and particle–wall interactions, the basic equations of the DEM were used, which follow a Lagrangian approach. The simulation software LIGGGHTS^®^ (version 4.1.0) was used for this purpose. In the DEM, the trajectories of all individual discrete particles were followed, and their velocity and acceleration were determined by deriving the trajectory curve according to time. According to Newton’s equation of motion, the product of mass $m_{i}$ and acceleration ${\ddot{x}}_{i}$ of a particle i is equal to the sum of all acting forces ${\vec{F}}_{i}$ (Equation (1)). The force and moment balances are shown in Equations (1) and (2), respectively:

(1) $$m_{i}{\ddot{x}}_{i}={\vec{F}}_{i,n}+{\vec{F}}_{i,t}+{\vec{F}}_{i,f}$$

(2) $${\vec{I}}_{i}\frac{d\omega_{i}}{dt}={\vec{R}}_{i}\times {\vec{F}}_{i,t}+{\vec{T}}_{i,r}$$

where ${\vec{F}}_{i,n}$ is the particle–particle contact force in the normal direction, ${\vec{F}}_{i,t}$ is the particle–particle contact force in the tangential direction, ${\vec{F}}_{i,f}$ is the resistance force, which acts on the particles through the surrounding fluid, ${\vec{I}}_{i}$ is the moment of inertia and $\omega_{i}$ is the angular velocity, ${\vec{T}}_{i,r}$ describes an additional torque that can be used to simulate nonspherical particles [38], and ${\vec{R}}_{i}$ is the position vector of the contact point of two particles. In addition to the forces mentioned here, other forces, such as electrostatic or magnetic forces, can also be taken into account. Furthermore, the interparticular interaction is described by means of contact models. To calculate the contact forces, the linear spring-damper model is often useful. The normal force is thus composed of the spring and damper forces:

(3) $${\vec{F}}_{i,n}=-k_{n}\cdot \delta_{n}+c_{n}\cdot \Delta {\vec{u}}_{n}$$

where $k_{n}$ and $c_{n}$ represent the spring and damper coefficients, $\Delta {\vec{u}}_{n}$ describes the relative velocity of the particle in the normal direction, and $\delta_{n}$ describes the virtual overlapping of two particles. In a real collision of two particles, a part of the energy is converted into heat by friction and plastic deformation. This dissipation energy is represented by damping elements with the damping coefficients in the normal direction $c_{n}$ in the model. According to Hertz and Mindlin [41,42,43], $k_{n}$ and $c_{n}$ can be calculated from the material properties in the normal (index n) and tangential directions (index t). The tangential force is limited by the static friction force and consists of the shearing force and the damping force in the tangential direction:

(4) $${\vec{F}}_{t}=min\left\{\left|k_{t}\cdot \overset{t}{\underset{t_{c}}{\int }}\Delta {\vec{u}}_{t}\cdot dt+c_{t}\cdot \Delta {\vec{u}}_{t}\right|,\;\left|\mu \cdot {\vec{F}}_{n}\right|\right\}$$

In Equation (4), $t_{c}$ is the time when particles come in contact, $\Delta {\vec{u}}_{t}$ is the tangential component of the relative velocity, and μ stands for the coefficient of friction. In addition to the balance of forces and moments, the energy equation for discrete particles must also be considered for modeling the melting behavior. The basic equations for the description of heat conduction in LIGGGHTS^®^ are originally based on the investigations of Chaudhuri [44]. Accordingly, the heat flux ${\dot{q}}_{i,j}^{cond}$ between the individual particles i and j due to heat conduction is calculated as follows:

(5) $${\dot{q}}_{i,j}^{cond}=h_{i,j}\cdot \Delta T_{i,j}$$

where $T_{i,j}$ corresponds to the particle temperature and $h_{i,j}$ to the interparticular heat transfer coefficient, which is calculated according to Equation (6) from the thermal conductivity coefficient $k_{i,j}$ and the contact area $A_{con}$ of the colliding particles:

(6) $$h_{i,j}=\;\frac{4k_{i}k_{j}\;}{k_{i}+k_{j}}\cdot {\left(A_{i,j}\right)}^{1/2}$$

where $k_{i,j}$ is surface-independent and has therefore the dimension $\frac{J}{K\cdot s\cdot m}$, whereas $h_{i,j}$ has the dimension $\frac{J}{K\cdot s}$. The temporal temperature development for a discrete particle $\frac{dT_{i}}{dt}$ can be calculated according to the following energy balance:

(7) $$m_{i}c_{i}\frac{dT_{i}}{dt}=\;\overset{}{\underset{i-j}{\sum }}{\dot{q}}_{i,j}+\;{\dot{q}}_{src/sink}$$

where $c_{i}$ corresponds to the specific heat capacity and $m_{i}$ is the mass of the particle. The first term on the right side of Equation (7) represents the heat flux due to particle interaction, and the second term describes heat sources and sinks. The conventional DEM is based on ideal spherical particles, which is a valid assumption for most applications.

In a real melting process, not only particle–particle and outer particle–wall heat transfer is of special interest. The rate of heat transfer $\dot{q}$ due to convective heat transfer through the surrounding fluid to the particle must also be considered and can be calculated according to Equation (8):

(8) $$\dot{q}=h_{conv}\cdot A\cdot \left(T_{\infty }-T_{i}\right)$$

where $T_{\infty }$ is the temperature of the surrounding fluid and $T_{i}$ is the temperature of the particle; $h_{conv}$ represents the heat transfer coefficient and is not a material parameter. It is generally difficult to determine for bulk materials and is calculated in LIGGGHTS^®^ using the Nusselt number. More about the convective heat transfer model used can be found in Li and Mason [45].

### 2.2. Extended CFD-DEM Approach

**Figure 1:**
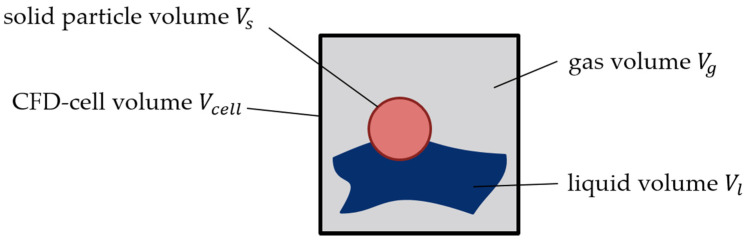
Schematic two-dimensional (2D) representation of the volume fractions at the coexistence of three phases in one CFD cell.

For the CFD-DEM simulation, the software CFDEMCoupling^®^ was used, which is an extension of OpenFOAM^®^ (version 5.x), enabling coupled simulations with LIGGGHTS^®^. Before the advanced CFD-DEM conservation equations are presented, a schematic 2D consideration of the coexistence of three phases in any finite volume element (CFD cell) is necessary (Figure 1).

The three phases can be divided into the solid state (plastic granules here represented by discrete particles), the liquid state, which consists of the melt that forms during the melting process, and the gas phase represented by the surrounding air.

The so-called voidfraction ε at a CFD cell is defined as the ratio between the volume occupied by the fluid $V_{f}$ (gas phase plus liquid phase) and the total volume of the CFD cell $V_{cell}$:

(9) $$\varepsilon =\frac{V_{f}}{V_{cell}}=1-\varepsilon_{s}$$

where $\varepsilon_{s}$ is the solid fraction, defined as the ratio between the volume occupied by the solid fraction $V_{s}$ and the volume of the CFD cell. Hence, ε assumes to be 1 if there are no particles in the CFD cell and strives towards the value of 0 if more particles are in the CFD cell. 

The total fluid volume $V_{f}$ results from the sum of the volumes of the individual phases:

(10) $$V_{f}=V_{l}+V_{g}$$

These must be calculated partially. The respective phase fraction $\alpha_{i}$ results from the ratio of the volume of the respective phase $V_{i}$ to the total fluid volume $V_{f}$:

(11) $$\alpha_{l}=\frac{V_{l}}{V_{f}}$$

(12) $$\alpha_{g}=\frac{V_{g}}{V_{f}}$$

The following volume balance can be drawn up:

(13) $$V_{s}+V_{f}\cdot \left(\alpha_{l}+\alpha_{g}\right)=V_{cell}$$

By diving both terms of the equation by $V_{cell}$, the following equation is obtained:

(14) $$\varepsilon_{s}+\varepsilon \cdot \left(\alpha_{l}+\alpha_{g}\right)=1$$

In light of this consideration, the conservation equation for phase fractions can now be established in the form of a transport equation. The transport of the phase fractions $\alpha_{l}$ and $\alpha_{g}$ takes place via the flow velocity ${\vec{u}}_{f}$. In addition, the temporal mass change ${\dot{s}}_{melt}$ must be considered due to the phase transition during melting. The mass conservation equations are then shown as follows:

(15) $$\frac{\partial \left(\rho_{l}\alpha_{l}\right)}{\partial t}+\nabla \cdot \left(\rho_{l}\alpha_{l}{\vec{u}}_{f}\right)={\dot{s}}_{melt}$$

(16) $$\frac{\partial \left(\rho_{g}\alpha_{g}\right)}{\partial t}+\nabla \cdot \left(\rho_{g}\alpha_{g}{\vec{u}}_{f}\right)=0$$

Since the mass of the gas phase is preserved and is not converted, the right side of Equation (16) is zero. The conservation of momentum (see Equation (17)) is analogous to an unresolved CFD-DEM approach according to Kloss et al. [38]. With an unresolved CFD-DEM approach, it must be ensured that each CFD cell in the computational area provides at least the volume of one spherical particle.

(17) $$\frac{\partial \left(\rho_{f}\alpha_{f}{\vec{u}}_{f}\right)}{\partial t}+\nabla \cdot \left(\rho_{f}\alpha_{f}{\vec{u}}_{f}{\vec{u}}_{f}\right)=\nabla \cdot \left(\alpha_{f}\underline{\tau }\right)-\alpha_{f}\nabla p+\alpha_{f}\rho_{f}\vec{g}+\sigma \kappa \frac{\nabla \left(\alpha_{l}\right)}{\left|\nabla \left(\alpha_{l}\right)\right|}-K_{sf}\left({\vec{u}}_{f}-\langle {\vec{u}}_{p}\rangle \right)$$

In Equation (17), $\alpha_{f}$ is the fluid volume fraction occupied by the liquid–gas phase, ∇p is the pressure gradient, $\underline{\tau }$ is the stress tensor, $\alpha_{f}\rho_{f}\vec{g}$ is the gravitational term, σ indicates the surface tension between the gas and liquid states, κ is the curvature of the gas–liquid interphase, and the expression $K_{sf}$ describes the exchange of moments between the fluid and solid phases taking into account the particle velocity ${\vec{u}}_{p}$. In the past, there have been various approaches to modeling the momentum exchange term [46,47,48]. A combination according to Gidaspow et al. [49] of the Ergün equation [50] for fluid volume fractions $\varepsilon_{f}$ of ≤0.8 and a model according to Wen and Yu [51] for $\varepsilon_{f}>0.8$ has become generally accepted. For further details, please refer to Kloss et al. [38].

The transport of the fluid phase volume fraction $\alpha_{f}$ is based on the VOF method [39,40] and is described by Equation (18):

(18) $$\frac{\partial \left(\alpha_{l}\right)}{\partial t}+\nabla \cdot \left(\alpha_{l}\alpha_{g}{\vec{u}}_{f}\right)-\nabla \cdot \left(\alpha_{l}\left(1-\alpha_{l}\right)\cdot {\vec{u}}_{c}\right)=-\alpha_{l}\frac{\partial \left(\alpha_{g}\right)}{\partial t}$$

where the source term on the right side accounts for the fluid displaced by the particles. According to Rusche [52], the compression term including the compression velocity ${\vec{u}}_{c}$ leads to a sharper interphase between the two different fluid phases. 

### 2.3. Novel Melting Model for Spherical Solids

In order to describe a phase transition from the solid state to melt and the resulting change of the mass over time, a novel melting model was developed and implemented into the simulation environment CFDEMCoupling^®^. This melting model mathematically describes the unsteady heating of a discrete spherical particle using the unsteady heat conduction equation. In reality, the heating of a plastic granule takes place from outside to inside. This results in an inhomogeneous temperature field. In the model presentation, a spherical particle is therefore discretized for the mathematical description of the temperature distribution into “shells”. One spherical particle is therefore composed of any number of shells.

**Figure 2:**
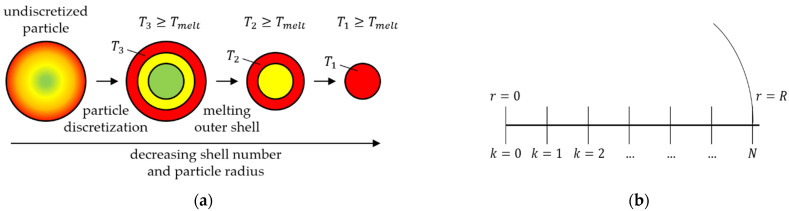
(**a**) Model presentation: heating of a spherical particle consisting of three shells; (**b**) discretization of a spherical particle with k=0 to N shells over the entire particle radius R.

As an example, in Figure 2a, it is assumed that a particle is composed of three shells. If a particle is heated until the temperature of the outermost shell ($T_{3}$) reaches the specified melting temperature ($T_{melt}$), the outermost shell enters the melt phase. Two more shells then remain, which also enter the melt phase as soon as their temperatures ($T_{2}$, $T_{1}$) exceed the melting temperature.

It should also be noted that the melting of the shells is accompanied by a reduction of the particle radius. As soon as the particle radius falls below a specified minimum value $r_{min}$, the particle is considered completely molten.

The unsteady temperature distribution within a shell is calculated according to Equation (19) using the one-dimensional unsteady heat conduction equation in spherical coordinates, assuming that only a temperature gradient is formed in the radial direction r:

(19) $$\rho_{s}c_{p}\frac{\partial T}{\partial t}=\lambda_{s}\left(\frac{2}{r}\frac{\partial T}{\partial r}+\frac{\partial^{2}T}{\partial r^{2}}\right)$$

For the calculation of the temperature distribution, the thermal conductivity of the particle $\lambda_{s}$, its density $\rho_{s}$, and specific heat capacity $c_{p}$ must therefore be known. In a good approximation, these material properties can be regarded as temperature-independent in the respective temperature range. To calculate the temperature distribution within a shell k for N shells (Figure 2b), Equation (19) is linearized using a backward Euler method:

(20) $$\frac{T_{n+1,\;k}-T_{n,\;k}}{\Delta t}=D\left(\frac{T_{n+1,\;k+1}-T_{n+1,\;k-1}}{r\Delta r}+\frac{T_{n+1,\;k+1}-2T_{n+1,\;k}+T_{n+1,\;k-1}}{\Delta r^{2}}\right)$$

where n corresponds to the discretized time step, the term $\frac{\lambda_{s}}{\rho_{s}c_{p}}$ was combined to form the diffusion coefficient D. For the solution of the one-dimensional unsteady heat conduction equation, a boundary condition for the calculation of the heat flux q with the environment at the position r=R is furthermore given as:

(21) $$q{|}_{r=R}=-\;\lambda_{s}\nabla T$$

With the equations listed so far, the temperature distribution in a particle can be calculated that exchanges heat with other particles in the environment with a wall or with a surrounding fluid. For the melting process, the change of the particle radius and especially the time-varying molten mass must also be taken into account.

The melting rate is calculated according to Equation (22) and is transferred for the CFD calculation to Equation (15):

(22) $${\dot{s}}_{melt}=\frac{m_{melt}}{\Delta t}=\frac{-\left(-\lambda_{s}\cdot C+\beta \right)}{L}\;\cdot A$$

where L is the mass related melting enthalpy, which is determined experimentally by means of differential scanning calorimetry (DCS), β describes the heat flux through the particle surface. In the absence of sources or sinks, this term corresponds to the term $q{|}_{r=R}$ in Equation (21), related to the surface of the sphere, A is the surface area of the discrete spherical particle through which the heat exchange occurs, and C is equal to the temperature gradient between two shells and described as:

(23) $$C=\frac{T_{n,\;k}-T_{n-1,\;k}}{\Delta r}$$

The reduction of the particle radius due to melting is described by the term Δr similar to Equation (24):

(24) $$\Delta r=\frac{R}{N-k}$$

where R is the initial radius of the particle and N−k describes the decreasing number of shells within a particle depending on time and temperature.

### 2.4. Calculation of Frictional Heating

For smooth and spherical particles, the dissipation and frictional heat $P_{diss}$ in the normal n and tangential t directions can be calculated via the respective damping force F and the relative velocity component $u_{n,t}$ at the contact point:

(25) $$P_{diss}=F_{n,t}\cdot u_{n,t}$$

In case of contact between two particles, the power dissipated is distributed among the two as follows [53]:

(26) $$P_{diss,\;1}=\frac{1}{2}\cdot \left(\frac{k_{1}}{k_{1}+k_{2}}\right)\cdot P_{diss}$$

(27) $$P_{diss,\;2}=\frac{1}{2}\cdot \left(\frac{k_{2}}{k_{1}+k_{2}}\right)\cdot P_{diss}$$

where k represents the thermal conductivity of the generic particle. The model employs Equations (25)–(27) to calculate the respective heat flux for each individual particle.

## 3. Melting Apparatus and Simulation Model

### 3.1. Principles and Functionality

In order to validate the melting model presented in Section 2.3, a novel melting apparatus was set up and put into operation within the scope of this work. The apparatus is based on the work of Chung 2010 [54] and is intended to determine the melting performance of a real single-screw extruder by scaling up the screw diameter. The extruder conditions that contribute to the melting process are imitated in the melting apparatus. Similar devices are called “screw simulator” in the literature [54].

**Figure 3:**
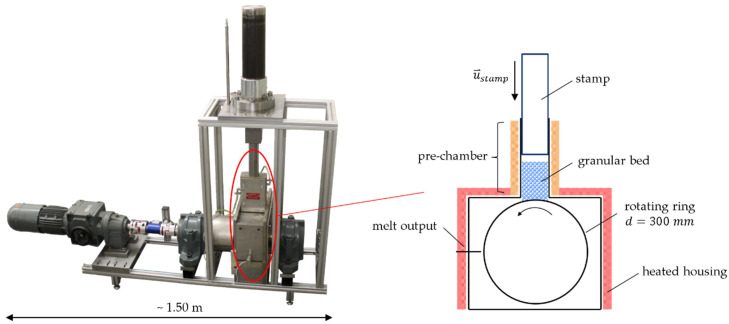
Melting apparatus and principle of operation.

The schematic sketch in Figure 3 illustrates how it works. A stamp with the cross-sectional dimensions of 50 mm × 30 mm, driven by a hydraulic system, compresses in a prechamber by its axial movement the previously inserted and weighed quantity of plastic granule. The force of the stamp is adjustable, and the axial position is detected by a distance-measuring system. The maximum length covered by the stamp is 300 mm.

The prechamber can be heated up to 200 °C, but a significantly lower temperature is set during the experiments to prevent premature melting of the granules. The actual melting is performed by the rotating ring which is heated to 200 °C by means of a slip ring and is located directly downstream of the prechamber. The ring’s shape can be designed as desired to investigate geometric factors influencing the melting behavior. For example, a smooth surface or an axially or helically grooved surface can be used. This means that the rotating ring takes over the task of the barrel in single-screw extrusion.

The ring has a diameter of 300 mm and is mounted on a rotating shaft driven by an electric motor. The shaft is supported against axial displacement by spherical roller bearings. The surrounding housing is heated to ensure almost adiabatic conditions. The melt film that forms on the surface of the ring can be removed with a melt scraper. The mass of plastic granules molten over time is then weighed.

Thus, by means of the melting apparatus, the melting rate can be determined experimentally under different process conditions such as speed and temperature for different materials. For the digital signal processing and output of the data a user interface within the used software LabVIEW^®^ [55] was developed.

### 3.2. Process Window

The melting rate was the target value of the experimental investigation, as it served as a comparative value for the evaluation of the melting model. As a parameter, the rotational speed was varied according to the overview in Table 1. All the other variables such as the stamp the force with which the granules were compacted and the temperatures in the housing in a prechamber and in a ring were kept constant during the test. The rotating ring was grooved. The 17 grooves had a width of 5.5 mm. For each operating point, the melting rate was determined six times to ensure sufficient accuracy. The procedure for the experimental determination of the melting rate can be described as follows:

- Predrying of the plastic;
- Heating up the melting apparatus;
- Filling the prechamber with a defined amount of granules when the stamp is moved out;
- Control of the stamp and start of the drive which sets the ring in a rotational movement; and
- Removal of the forming melt film and stopping the time as soon as a steady state is reached.

### 3.3. Materials and Characterization

A polypropylene, Moplen RP 210G [56] by LyondellBasell, Rotterdam, Netherlands, was used for the tests. The mass of the granules filled into the prechamber was always 100 g. In the following, the material used was referred to as PP.

For the performance of CFD-DEM simulations, a large number of material parameters were required. Since the melting process was simulated in this work, thermodynamic parameters were required in addition to the mechanical parameters that are usually necessary for a pure DEM simulation. To achieve reliable simulation results, these should be determined as accurately as possible and included into the simulation model.

Table A1 (Appendix A) gives an overview of some important material parameters and the respective determination method or equipment used. For example, to determine Young’s modulus, tensile bars according to ISO 527-2:2012—Type 1A were injection molded on an Arburg Allrounder 320 S from the manufacturer Arburg, Lossburg, Germany. Furthermore, the particle size distribution was determined with a modern scanner and was also considered in the simulations.

### 3.4. CFD-DEM Modeling of the Melting Apparatus

**Figure 4:**
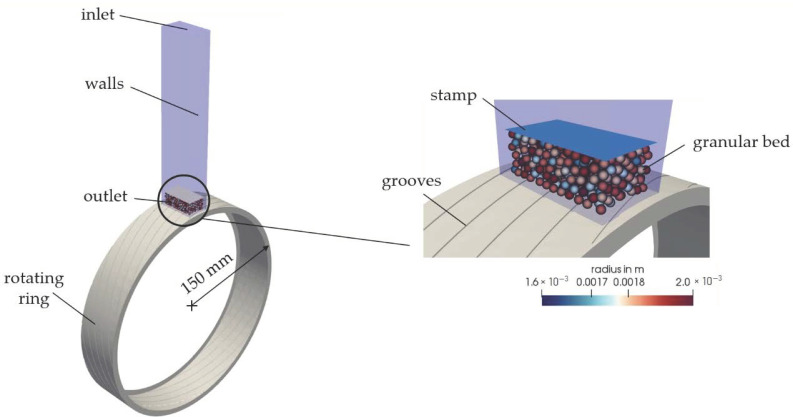
CFD-discrete element method (DEM) model of the melting apparatus. The CFD domain is shown in blue.

The melting apparatus was modeled according to the experimental setup. In general, a CFD-DEM simulation should be limited to the respective domains. With the apparatus shown in Figure 3, the melting process is expected to take place in the prechamber. The housing surrounding the rotating ring plays a subordinate role, since the largest part of the melt that forms is discharged via a melt scraper. For this reason, it is sufficient to limit the modeling and simulation of the apparatus to the prechamber, the stamp, and the grooved ring (see Figure 4). This also saves computing time, because the computing area is considerably reduced.

For the calculation, a basic distinction must be made between the types of computational meshes. In CFD simulations volume meshes were required, whereas surface meshes were required in DEM simulations. Both grid types were generated with the help of ANSYS ICEM CFD^TM^ [57].

On the DEM side, the compaction of a granular bed was modeled by the axial movement of the stamp over a simple rectangular surface. The cross-section corresponded to the dimensions of the stamp of the experimental setup. Furthermore, the rotation of the grooved ring and the resulting force transmission to the particles were taken into account. The grooves were completely resolved. The stamp and the grooved ring were imported into LIGGGHTS^®^ as surface meshes in the Standard Triangulation Language (STL).

The CFD domain represents the prechamber and the rotation of the grooved ring. This is shown in blue in Figure 4. As there is no flow, it only represents an air-filled volume in this case. However, this volume is essential, under which the calculation of the melting rate is possible according to the presented CFD-DEM equations.

The temperature boundary conditions were specified both in the CFD and in the DEM according to the experiments (see Table 1).

**Figure A1:**
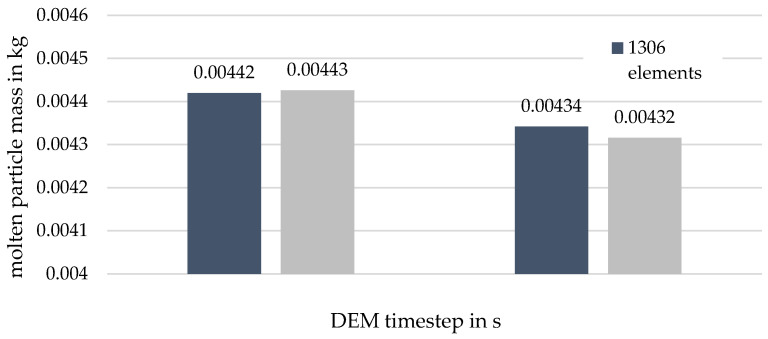
Molten particle mass depending on the number of calculation elements and the selected DEM time step.

As explained in Section 2.2, the CFD computational mesh generation in an unresolved CFD-DEM approach represents a special challenge, since there is a dependency between the volume of the CFD cell and the particle volume. The cell size is thus limited by the particle diameter. Therefore, a computational mesh study was performed in advance to ensure mesh and time convergence. More detailed explanations including the results can be found in Figure A1 (Appendix B). The software ParaVIEW [58] was used to visualize the simulation results.

## 4. Results and Discussion

The discussion of the results focuses on the evaluation of the melting rate from the experiment and the simulation and their comparison. First, in Section 4.1, the influence of the number of shells on the simulation result is highlighted. Afterwards, in Section 4.2, the melting rates obtained using the new melting model are compared and discussed with experimental results of the measuring apparatus.

### 4.1. Influence of the Number of Shells

After the successful implementation of the melting model into the CFDEMCoupling^®^ framework, the first issue to be clarified is how the number of shells per DEM particle influences the melting behavior. For this purpose, CFD-DEM simulations using PP were performed for the melting apparatus and for a number of shells (see Figure 2) ranging from 1 to 100. In order to save computing time, the rotational speed was set to zero. The stamping force was 100 N. The percentage of the molten mass ξ can then be determined by evaluating the simulations as follows:

(28) $$\xi =\left(\frac{m_{total}}{m}-1\right)\cdot 100$$

where $m_{total}$ is the total mass specified in the simulation at time 0 and it was deliberately set to 12 g in the simulation to reduce computational time, which was reasonable, since the change of mass per time was of interest; m stands for the value of the mass at any further time and was returned from the simulation.

**Figure 5:**
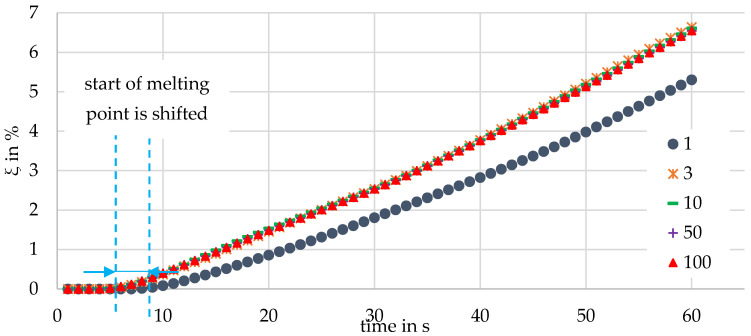
Percentage of molten mass as a function of the number of shells applied over time (stamp force = 100 N, rotational speed = 0 min^−1^).

Figure 5 shows the course of ξ for different numbers of shells over a simulation time of 60 s. A shell number of one corresponds to the case that the whole particle was considered as molten when the particle temperature exceeded the melting temperature, i.e., no discretization of the particle took place.

All the characteristic curves are similar: Up to approximate 10 seconds, the particles were heat up and ξ was approximately 0. In the further course, ξ rose continuously. It can be observed that by increasing the number of shells from one to three, the start of melting was shifted. This can be explained as follows: It took less time to melt the outermost shell of a particle with three shells than a complete, nondiscretized particle (shell number = 1). The outermost shell of a particle with three shells thus melted earlier, which was reflected in the increase in the molten mass.

This means that three shells melted up more in percentage terms and the gradient $\frac{d\xi }{dt}$ was slightly steeper. If the number of shells was further increased to 10, 50, and 100, no significant influence can be seen, demonstrating that the discretization achieved sufficient accuracy with three shells.

### 4.2. Comparison of the Calculated and Measured Melting Rates

**Figure 6:**
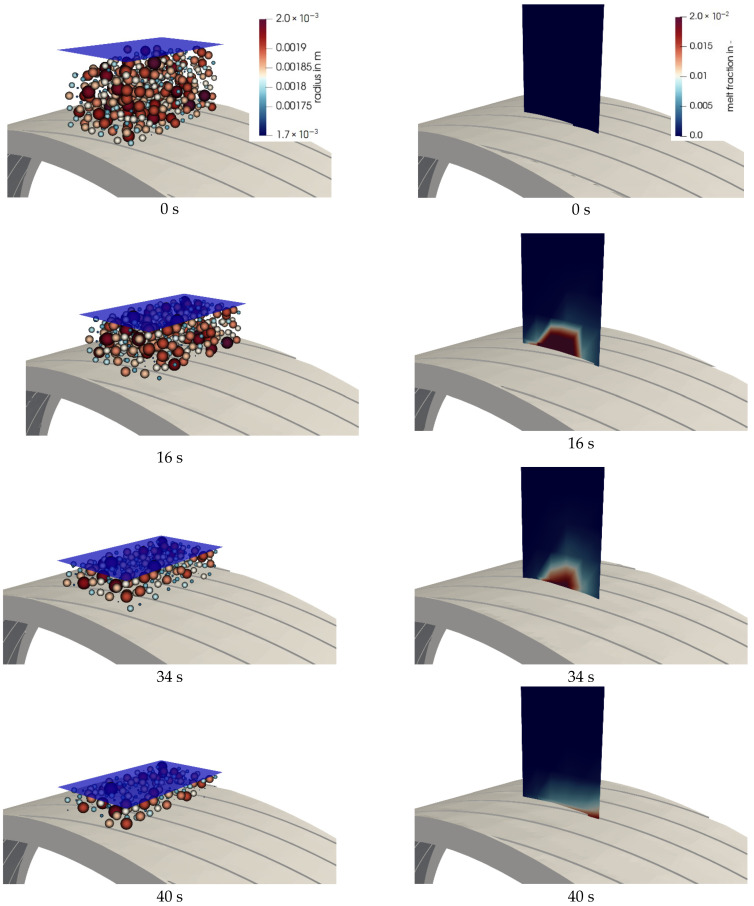
(**Left column**) particle radius shrinks and stamp compresses particles during melting in the DEM domain; (**right column**) melt fraction formation in a prechamber in the CFD domain. Rotational speed: 3 min^−1^; simulation time: from 0 to 40 s.

First, the CFD-DEM simulation results based on the implemented melting model are illustrated. The left column in Figure 6 shows the melting phenomena in the DEM domain of the melting apparatus. The stamp compresses the particle bed in the prechamber with a force of 1000 N and presses the particles onto the grooved ring, which rotated at a speed of 3 min^−1^. In the left column, the reductions of the particle radius and particle number due to phase transition are shown. The right column in Figure 6 shows the melting phenomena in the prechamber and the grooved ring in the CFD domain.

For reasons of clarity, a plane was defined exactly in the middle through the prechamber. At the same time in the process as the DEM simulation, the formation of melt in the prechamber can be observed on the basis of the corresponding phase fraction.

At time zero, the particles were present in their natural size distribution in a loose bulk. At this time, no melt formed in the CFD domain, as can be seen in the right column of Figure 6. After 16 s, the particles were already compressed and partially molten. This can be seen from the fact that the volume fraction of the melt immediately above the grooved ring was greater than zero. The melting process therefore took place as expected on the heated grooved ring. This is where the highest temperature occurred. The melting process was also greatly enhanced by the frictional power of the grooved ring.

In the further course of the coupled simulation, the number of particles decreased steadily, and melt continued to form, which was afterwards transported. Similar behavior was observed for the other considered operating points at rotational speeds of 2 and 3 min^−1^.

**Figure 7:**
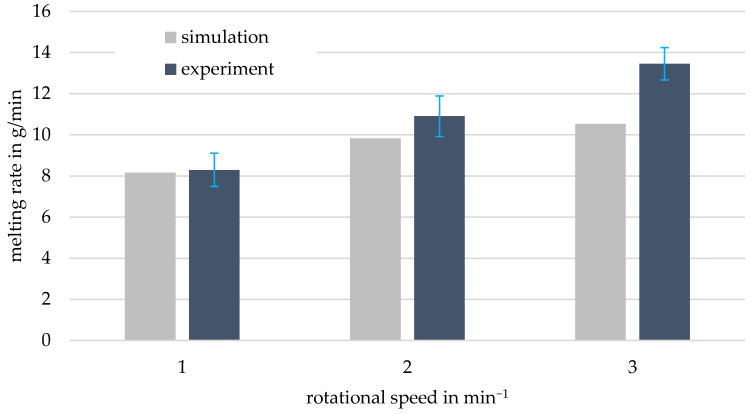
Calculated and experimentally measured melting rates for different rotational speeds at a stamp force of 1000 N and a ring temperature of 200 °C.

After the melting process was illustrated, a comparison between the results of simulation and experiment was drawn. Figure 7 shows the measured melting rates for three rotational speeds compared to the simulation results.

The melting rate increased with increasing speed under otherwise constant conditions. This observation is in line with the existing state-of-the-art investigations [31,59] and thus confirmed the correct functioning of the melting apparatus. It can be seen that the simulated melting rates were lower than the experimental values for all the rotational speeds.

The deviations of simulation results from the experiment were between 1.68% at 1 min^−1^ and 21.71% at 3 min^−1^. The deviations from the experiment can be attributed to the following unavoidable simplifications of the melting model and the limits of the DEM:

- The melting model was set up for ideal spherical particles. However, the PP used in the experiment had an ellipsoidal granular form.
- In the experiment, granules were deformed by the stamp force and the rotation of the grooved ring, favoring the melting process. However, the DEM cannot completely map this kind of deformation of the particles. That is why the simulation values were lower than the experimental results.
- The Young’s modulus, which has a considerable influence on the melting behavior, was in reality temperature-dependent, but was assumed to be constant in the DEM. A temperature-dependent implementation of the Young’s modulus is not possible at present, as the Rayleigh time and the numerical stability of the simulation are directly influenced.

The deviations may seem large at first glance. However, the results achieved with the new modeling approach are very promising and overall in good agreement with experimental results.

In regard of the evaluation of the results, it is even more important to mention that no parameter or model adjustments were undertaken in the coupled simulation of the melting apparatus. Only experimentally determined substance data were used instead.

## 5. Conclusions and Outlook

Within the scope of this study, a novel melting model was developed. This describes the phase transition from the solid state of the plastic to the melt state by means of the CFD-DEM approach for the first time. The melting model considers the temperature change due to heat conduction, convection, and frictional heating. For the validation of the simulation results, a melting apparatus was set up and put into operation. Melting experiments were performed to determine the melting rate of PP. In parallel, the experimental setup was modeled. The influence of the number of shells on the particles’ melting behavior was drawn. Considering the necessary simplifying assumptions in the model and limitations in the CFD-DEM approach, a comparison between the simulation and the experiment showed a very good agreement without any adjustment of the parameters. 

To confirm the transferability of the melting model, the experiments were extended to further experimental parameters and materials. In particular, the stamp force, the ring temperature, and the geometry of the ring should be varied.

As a next step, the model should be transferred to the single-screw extrusion. The modeling of the extrusion is currently proving to be much more challenging, since dynamic computational meshes must be used on the CFD side in order to correctly represent the conveying process of granules. However, a CFD-DEM simulation model of the extrusion has been successfully created.

## Figures and Tables

**Table 1:** Overview of the testing parameters applied.

Parameter	Value
rotational speed	1, 2, and 3 min^−1^
housing temperature	200 °C
ring temperature	200 °C
prechamber temperature	130 °C
stamp force	1000 N
rotating ring	helically grooved

## Data Availability

Not applicable.

## Appendix A. Overview of Important Required Material Data

**Table A1:** Overview of the required parameters for the CFD-DEM simulation and determination method.

Mechanical and Other Properties	Method of Determination and Equipment Used
Young’s modulus	Tensile test acc. to DIN EN ISO 527 (temperature-dependent)
Coefficient of friction	Literature value
Poisson’s ratio	Literature value
Coefficient of restitution	Universalprüfmaschinen ZPM1455, Zwick GmbH, acc. to ISO DIN EN ISO 527
Density (solid state)	Data sheet PPRP210G
particle size distribution	High-resolution scanner Epson V850 pro, Epson K.K.
**Thermodynamic properties**	
Specific heat capacity	Differential scannig calorimetry acc. to DIN EN ISO 11357-4
Thermal diffusivity	Laser flash analysis
Latent heat	Differential scannig calorimetry acc. to DIN EN ISO 11357-4
Density (liquid state)	Acc. to DIN EN ISO 1183-1

## Appendix B. Mesh Convergence Study

With a coupled CFD-DEM simulation, the sole analysis of the mesh influence and the given CFD time step on the simulation results was not as sufficient as with a pure CFD simulation. In addition, the influence of the selected DEM time step must be considered, since the solver does not adapt the time step automatically. For this investigation, the melting apparatus was simulated.

To save computing time, the simulation time was set to twelve seconds, whereby the initialized mass of the particles was 0.012 kg. The molten mass was evaluated for two different computational meshes and DEM time steps (see Figure A1).

Figure A1 shows that the mesh and time step convergence was given for the coarser computational mesh with 7.232 elements at a DEM time step of 5 × 10^−6^ s. The CFD time step was chosen two orders of magnitude higher than the DEM time step and was thus 5 × 10^−4^ s. The average relative deviation due to the grid influence was less than 1%. Within different time steps, the average relative deviation was 2.17%. It was therefore sufficiently small to confirm the time-step convergence.
